# Cardioprotective Effect of Paeonol and Danshensu Combination on Isoproterenol-Induced Myocardial Injury in Rats

**DOI:** 10.1371/journal.pone.0048872

**Published:** 2012-11-06

**Authors:** Hua Li, Yan-Hua Xie, Qian Yang, Si-Wang Wang, Bang-Le Zhang, Jian-Bo Wang, Wei Cao, Lin-Lin Bi, Ji-Yuan Sun, Shan Miao, Jing Hu, Xuan-Xuan Zhou, Peng-Cheng Qiu

**Affiliations:** 1 Institute of Materia Medica, School of Pharmacy, The Fourth Military Medical University, Xi'an, People's Republic of China; 2 Department of Pharmaceutics, School of Pharmacy, The Fourth Military Medical University, Xi'an, People's Republic of China; Thomas Jefferson University, United States of America

## Abstract

**Background:**

Traditional Chinese medicinal herbs Cortex Moutan and Radix Salviae Milthiorrhizaeare are prescribed together for their putative cardioprotective effects in clinical practice. However, the rationale of the combined use remains unclear. The present study was designed to investigate the cardioprotective effects of paeonol and danshensu (representative active ingredient of Cortex Moutan and Radix Salviae Milthiorrhizae, respectively) on isoproterenol-induced myocardial infarction in rats and its underlying mechanisms.

**Methodology:**

Paeonol (80 mg kg^−1^) and danshensu (160 mg kg^−1^) were administered orally to Sprague Dawley rats in individual or in combination for 21 days. At the end of this period, rats were administered isoproterenol (85 mg kg^−1^) subcutaneously to induce myocardial injury. After induction, rats were anaesthetized with pentobarbital sodium (35 mg kg^−1^) to record electrocardiogram, then sacrificed and biochemical assays of the heart tissues were performed.

**Principal Findings:**

Induction of rats with isoproterenol resulted in a marked (*P*<0.001) elevation in ST-segment, infarct size, level of serum marker enzymes (CK-MB, LDH, AST and ALT), cTnI, TBARS, protein expression of Bax and Caspase-3 and a significant decrease in the activities of endogenous antioxidants (SOD, CAT, GPx, GR, and GST) and protein expression of Bcl-2. Pretreatment with paeonol and danshensu combination showed a significant (*P*<0.001) decrease in ST-segment elevation, infarct size, cTnI, TBARS, protein expression of Bax and Caspase-3 and a significant increase in the activities of endogenous antioxidants and protein expression of Bcl-2 and Nrf2 when compared with individual treated groups.

**Conclusions/Significance:**

This study demonstrates the cardioprotective effect of paeonol and danshensu combination on isoproterenol-induced myocardial infarction in rats. The mechanism might be associated with the enhancement of antioxidant defense system through activating of Nrf2 signaling and anti-apoptosis through regulating Bax, Bcl-2 and Caspase-3. It could provide experimental evidence to support the rationality of combinatorial use of traditional Chinese medicine in clinical practice.

## Introduction

Ischemic heart disease (IHD) is the most serious crimes against human life, claiming 17 million lives per year [1]. Epidemiological studies indicate that IHD will constitute the major disease-burden worldwide by the year 2020 [2]. Myocardial infarction (MI) is a common and life-threatening manifestation of IHD. It occurs when myocardial ischemia surpasses the critical threshold level for an extended time, resulting in irreversible necrosis of the myocardium [3]. Myocardial infarction is invariably followed by numerous pathophysiological and biochemical alterations including hyperlipidemia, thrombosis, lipid peroxidation (LPO) and free radical damage etc., leading to qualitative and quantitative changes of myocardium [4]. It has also been suggested that oxidative stress produced by free radicals or reactive oxygen species (ROS), as evidenced by marked increase in production of lipid peroxidative products associated with decreased levels of antioxidants such as superoxide dismutase (SOD), catalase (CAT) and reduced glutathione (GSH), plays a major role in myocardial damage during MI [5].

Isoproterenol (ISO), a synthetic catecholamine and beta-adrenergic agonist, has been found to produce MI in large doses due to generation of highly cytotoxic free radicals, causing cardiac dysfunctions, increased lipid peroxidation, altered activities of cardiac enzymes and antioxidants, resulting in infarct like necrosis of the heart muscle [6]. The pathophysiological and morphological alterations of myocardium following ISO administration have been observed similar to those taking place in human MI [7]. Therefore, ISO-induced myocardial injury serves as a well standardized model to study the cardiac functions and beneficial effects of many drugs.

*Radix Salviae Milthiorrhizae* (root and rhizome of *Salvia miltiorrhiza* Bunge) and *Cortex Moutan* (root bark of *Paeonia suffruticosa* Andrew) are two traditional Chinese medicinal herbs widely used in China, Japan, Korea and India for their putative cardioprotective and cerebroprotective effects [8], [9]. In clinical practice, *Radix Salviae Milthiorrhizae* and *Cortex Moutan* are commonly used in combination, famous as *ShuangDan* prescription (SD), for the treatment of angina pectoris, myocardial infarction, and other cardiac symptoms [10]. It has been reported that SD significantly decreased infarct area of heart and CK, CK-MB levels in serum, modulated the expression of 23 oxidative stress related proteins in rats with coronary occlusion [11]. In order to evaluate the efficacy and to understand the mechanisms of *ShuangDan* prescription, two representative active principles of the two herbs, paeonol (Pae) from *Cortex Moutan* and danshensu (DSS) from *Radix Salviae Milthiorrhizae*, were selected for study.

**Figure 1:**
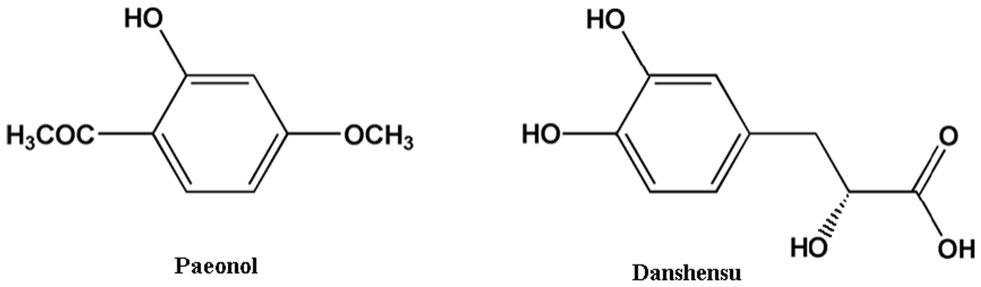
Chemical structures of paeonol (chemical name: 4-methoxy-2-hydroxyacetophenone), danshensu (chemical name: (2R)-3-(3, 4-dihydroxyphenyl)-2-hydroxypropanoic acid).

Paeonol (Pae, Fig. 1) is a phenolic acid compound extracted from *Cortex Moutan*. A number of studies have revealed that paeonol possesses many physiological activities, including vascular dilation [12], prevention of cardiovascular diseases [13] and improvement of arrhythmia [14]. According to several phytochemical studies, danshensu (DSS, Fig. 1) is abundant and structurally representative of the water-soluble active components of *Radix Salviae Milthiorrhizae* [15]. It has been demonstrated that danshensu reduces lipid peroxidation on mitochondrial membrane by scavenging free radicals, and inhibits permeability and transmission of mitochondrial membrane by reducing thiol oxidation [16], [17].

We previously reported the interesting pharmacokinetic phenomenon that the combination use of paeonol plus danshensu dramatically increased the concentration of danshensu in rat plasma and the concentration of paeonol in rat heart and brain [18]. Pharmacological studies also revealed that paeonol and danshensu in combination have synergistic protective effect on focal cerebral ischemia-reperfusion injury in rat, likely through improving the blood hemorrheology, decreasing oxidative injury and repairing the function of blood vessel endothelium [19].

However, there is little information on the cardioprotective effects of danshensu and paeonol combination *in vivo*. The present study was designed to evaluate the cardioprotective effects of danshensu and paeonol combination on ISO-induced myocardial injury and to understand their underlying mechanism.

## Materials and Methods

### Drugs and chemicals

Paeonol (99.0%, purity) and Danshensu (98.5%, purity) were provided by Dong Ke Madison Bio-tech. Co., Ltd. (Yanglin, Xi'an, China). Isoproterenol hydrochloride was purchased from Tokyo chemical industry Co., Ltd. (Kita-Ku, Tokyo, Japan). All other biochemical reagents and chemicals were of analytical grade.

### Animals and Ethics

Male Sprague-Dawley rats (220±20 g) were purchased from Experimental Animal Research Center, the Fourth Military Medical University (Xi'an, China). The animals were maintained in individually ventilated cages (IVC) system (12 h light/dark cycle, 20.3–23.1°C and 40–50% humidity) during the experiment cycle and fed with standard laboratory food and water ad libitum. There were no significant differences in the body weights of the treated rats when compared with control at the beginning of the study period. The treated rats did not offer any abnormal resistance to drug administration. The treatment schedule did not cause any change in food and water intake pattern. Experimental protocol (2011-0922-R) involving animals was reviewed and approved by the Institutional Animal Care and Use Committee of the Fourth Military Medical University.

### Induction of myocardial injury

Myocardial injury was induced in experimental rats by injection of 85 mg kg^−1^ of ISO daily for 2 consecutive days.

### Pilot study for dose fixation

Paeonol at the doses of 20, 40, 80 and 120 mg kg^−1^ day^−1^ and danshensu at the doses of 40, 80, 160 and 240 mg kg^−1^ day^−1^ were screened to determine the dose dependent effect of paeonol and danshensu combination in ISO-induced myocardial infarction in rats. The optimum dose exhibiting maximum cardioprotective effect during 21 days was evaluated by estimating electrocardiograph-abnormalities (determined as ST-segment), serum lactate dehydrogenase (LDH), creatine kinase (CK-MB), tissue lipid peroxidation and reduced glutathione content. Paeonol (80 mg kg^−1^ day^−1^, i.g.) and danshensu (160 mg kg^−1^ day^−1^, i.g.) were found to be most effective in functional recovery of biochemical alterations (data not shown). Hence these doses were selected for further evaluation (alone as well as in combination) in the present studies. Dose of isoproterenol was selected on the basis of reported literature [20].

### Experimental Design and Protocol

The experimental animals were divided into five groups of eight rats each.

Group I (Control group): control rats received 0.3% sodium carboxymethyl cellulose (CMC-Na) solution (2 ml kg^−1^ day^−1^, i.g.) for a period of 21 days and normal saline (1 ml kg^−1^, s.c.) on 20th and 21st day.

Group II (ISO group): rats received 0.3% CMC-Na solution for a period of 21 days and ISO (85 mg kg^−1^, s.c.) in normal saline on 20th and 21st day at an interval of 24 h.

Group III (Pae+ISO group): rats received paeonol (80 mg kg^−1^ day^−1^, i.g.) for a period of 21 days and ISO on 20th and 21st day.

Group IV (DSS+ISO group): rats received danshensu (160 mg kg^−1^ day^−1^, i.g.) for a period of 21 days and ISO on 20th and 21st day.

Group V (Pae+DSS+ISO group): rats received paeonol (80 mg kg^−1^ day^−1^, i.g.) and danshensu (160 mg kg^−1^ day^−1^, i.g.) for a period of 21 days and ISO on 20th and 21st day.

Paeonol and danshensu were suspended in 0.3% CMC-Na solution. Control and ISO treated group received equal quantity of vehicle.

At the end of the experimental period, rats were anesthetized with pentobarbital sodium (35 mg kg^−1^, i.p.), needle electrodes were inserted under the skin of the animals in lead II position. Electrocardiograph recordings were made using BL-420S Biologic Function Experiment system (Technology & Market Co., Ltd., Chengdu, China) and ST-segment elevation or depression (expressed in mv) in normal and experiment animals were considered.

After recording the ECG, blood was collected by abdominal aorta and allowed to clot for 1 h at room temperature. Serum was subsequently separated by centrifugation at 3500×*g* for 15 min and stored at −80°C for biochemical assays. After blood collection, rats were sacrificed by cervical decapitation. Heart tissue was excised immediately and rinsed in ice-cold normal saline, then homogenized by an IKA T10 Basic homogenizer (Staufen, Germany) in 0.05M ice-cold phosphate buffer (pH 7.4, 1∶10 *w/v*). The homogenate was centrifuged at 12000×*g* for 10 min at 4°C and the supernatant was stored at −80°C for the estimation of various biochemical parameters.

### Measurement of Myocardial Infarct Size

Direct triphenyl tetrazolium chloride (TTC) assay according to method of Lie et al. [21] was used to determine myocardial infarct size. In brief, the heart was transversely cut across the left ventricle, and sections of 2 mm to 3 mm thick were incubated in 1% TTC solution prepared in phosphate buffer (pH 7.4) for 30 min at 37°C, following which they were fixed with 10% formalin. The non-ischemic myocardium and viable ischemic myocardium were stained red, while the infarcted myocardium appeared pale grey or white. The slices were photographed using a digital camera, and the % infarction was analyzed using the computerized Image-Pro Plus 6.0 software (Media Cybernetics Inc, Silver Spring, MD, USA).

### Assay of cardiac marker enzymes

Activities of creatine kinase-MB (CK-MB), lactate dehydrogenase (LDH), aspartate aminotransferase (AST) and alanine aminotransferase (ALT) in the serum were measured using commercial kits (Biosino bio-technology and science Inc., Beijing, China).

### Estimation of cardiac troponin I

The levels of cardiac troponin I (cTnI) in serum were estimated using standard kit by enzyme-linked immunesorbent assay (Jiancheng bio-technology and science Inc., Nanjing, China).

### Estimation of lipid peroxidation products and antioxidants

Tissue lipid peroxide level in heart was determined as thiobarbituric acid reactive substances (TBARS) by the methods of Ohkawa et al. [22] using a reagent kit (BioAssay Systems, CA, USA). The activities of superoxide dismutase (SOD), catalase (CAT), glutathione peroxidase (GPx), glutathione reductase (GR) and glutathione-S-transferase (GST) were assayed by the methods of Oyanagui [23], Goth [24], Rotruck et al. [25], Pinto and Bartley [26] and Habig et al. [27], respectively. The level of reduced glutathione (GSH) was estimated by the methods of Ellman [28]. The content of protein in the heart homogenate was determined by the method of Bradford [29].

### Histopathological examination

After sacrifice, the cardiac apex was rapidly dissected out and washed immediately with ice-cold normal saline and fixed in 10% buffered formalin. The fixed tissues were embedded in paraffin, sectioned at 5 µm and stained with hematoxylin and eosin (H&E). The sections were examined under light microscope and photomicrographs were taken by a Zeiss Axioskop 40 photomicroscope at ×200 magnification.

### Immunohistochemistry

The paraffin sections, 5 µm thick, were deparaffinaged in xylene, dehydrated in a graded series of ethanol, subjected to antigen retrieval in phosphate-buffered saline (PBS) at 92–98°C for 5 min. The sections were allowed to cool down to the room temperature, washed in PBS, then incubated in 3% H_2_O_2_ in 0.01 M PBS for 10 min at room temperature, and in 5% BSA for 20 min at 37°C. Next, sections were incubated overnight at 4°C with primary antibody anti-Nrf2 (1∶100, Bioworld Technology, Inc., USA), anti-Bax (1∶200, Epitomics, Inc., USA), anti-Bcl-2 (1∶250, Cell Signaling Technology, Inc., USA) and anti-Caspase-3 (1∶200, Santa Cruz Biotechnology, Inc., USA). Negative controls included omission of primary antibody and use of PBS. Sections were then rinsed (PBS) and incubated for 1 h with specie peroxidase-conjugated secondary antibody (Boster Biotechnology, China). The reaction was visualized with a solution of diaminobenzidine (DAB). For quantification, integral optical density (IOD) of Nrf2, Bax, Bcl-2 and Caspase-3 staining were calculated with computerized Image-Pro Plus 6.0 software (Media Cybernetics Inc, Silver Spring, MD, USA).

### Statistical analysis

Results are shown as mean ± S.D.. One-way ANOVA was carried out, and the statistical comparisons among the groups were performed with Tukey's test using GraphPad Prism 5.0 statistical package program (GraphPad Software, Inc., La Jolla, CA, USA). *P*-values less than 0.05 were considered as statistically significant.

## Results

### Effect of paeonol and danshensu on heart weight, body weight and electrocardiograph parameters

**Figure 2:**
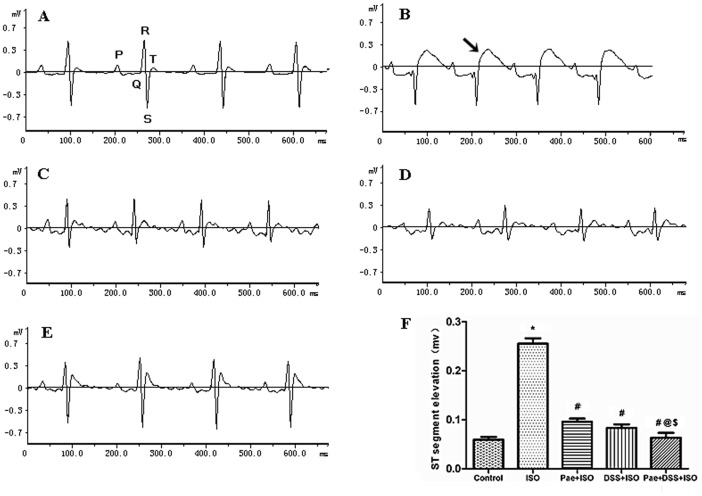
Representative electrocardiogram tracings [(A) control, (B) ISO, (C) Pae+ISO, (D) DSS+ISO and (E) Pae+DSS+ISO] and ST-segments changes (F) of control and experimental animals. Values are expressed as mean ± S.D. (n = 8). ^*^ *P*<0.001 vs. Control; ^#^ *P*<0.001 vs. ISO; ^@^ *P*<0.001 vs. Pae+ISO; ^$^ *P*<0.001 vs. DSS+ISO (one-way ANOVA). (ECG was recorded from II limb leads with recorder speed 50 ms/div).

The mean body weight of rats at the end of experiment period in all experimental groups had no significant change (Table 1), although ISO treated rats showed a slight reduction in body weight which was not significant. The heart weight and the ratio of heart weight to body weight were increased significantly (*P*<0.001) in ISO-administered groups when compared with normal control groups. Rats pre-co-treated with the combination of paeonol and danshensu showed a significant (*P*<0.001) reduction in the heart weight and the ratio as compared to ISO treated groups. ISO-administered rats showed marked ST-segment elevation (Fig. 2). Pretreatment with paeonol or danshensu in ISO-administered rats showed a significant (*P*<0.001) decrease in ST-segment as compared to ISO-administered rats. The pretreatment of paeonol and danshensu combination in ISO-administered rats showed a significant (*P*<0.001) decrease in ST as compared to ISO, Pae+ ISO or DSS+ISO treated groups.

**Table 1:** Effect of paeonol and danshensu on heart weight, body weight and heart weight/body weight ratio in rats.

Group(s)	Body weight (g) at the end of treatment period	Heart weight (g)	Heart weight/body weight ratio (%)
Control	268.2±12.25	0.808±0.053	0.302±0.028
ISO (85 mg/kg, s.c.)	256.8±12.1^ns^	1.164±0.057*	0.454±0.033*
Pae (80 mg/kg, i.g.)+ISO	258.7±9.31^ns^	0.995±0.053#	0.385±0.029#
DSS (160 mg/kg, i.g.)+ISO	259.2±8.78^ns^	0.967±0.047#	0.374±0.026#
Pae+DSS+ISO	272.3±11.12^ns^	0.864±0.055# @ ¥	0.317±0.022# @ ¥

Results are expressed as mean ± S.D. (n = 8).

* *P*<0.001 vs. Control;

# *P*<0.001 vs. ISO;

@ P<0.001 vs. Pae+ISO;

¥ *P*<0.01 vs. DSS+ISO,

$ *P*<0.001 vs. DSS+ISO;

ns–non significant (one-way ANOVA).

### Effect of paeonol and danshensu on myocardial tissue damage

**Figure 3:**
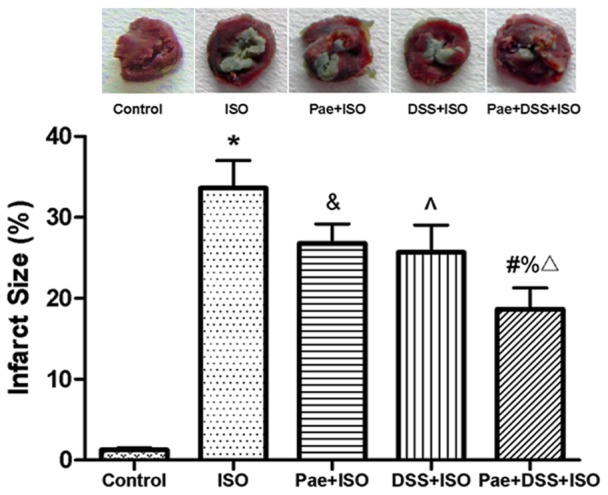
Myocardial infarct size in the control and experimental animals. Normal myocardium was stained red, while pale grey areas indicate infarct areas. Values are expressed as mean ± S.D. (n = 4). ^*^ *P*<0.001 vs. Control; ^&^ *P*<0.05 vs. ISO, ^∧^ *P*<0.01 vs. ISO, ^#^ *P*<0.001 vs. ISO; ^%^ *P*<0.01 vs. Pae+ISO; ^Δ^ *P*<0.05 vs. DSS+ISO (one-way ANOVA).

Representative illustrations of infarction size as stained TTC are shown in Fig. 3. While ISO administration indicated a large unstained area, however, the heart slice of the Pae+DSS+ISO-treated rats exhibited a major portion stained positively showing tissue viability. Fig. 3 also indicates the increased infarction area in ISO-administered group (33.63%), which was significant (*P*<0.001) reduced to 18.64% with the combined pretreatment of paeonol and danshensu.

### Effect of paeonol and danshensu on serum marker enzymes

As shown in Table 2, there was a significant (*P*<0.001) rise observed in the levels of diagnostic marker enzymes (CK-MB, LDH, AST and ALT) in the serum of group ISO-administered rats. During myocardial infarction condition, these enzymes were released into the blood stream. Pretreatment with paeonol and danshensu in combination showed a significant (*P*<0.001) reduction in the levels of all serum diagnostic marker enzymes compared to the ISO, Pae+ISO and DSS+ISO groups.

**Table 2:** Effect of paeonol and danshensu on serum marker enzymes in rats.

Group(s)	AST (IU/L)	ALT (IU/L)	CK-MB (IU/L)	LDH (IU/L)
Control	51.06±2.51	26.75±3.45	77.69±6.25	77.00±3.56
ISO (85 mg/kg, s.c.)	92.42±4.23*	58.38±2.85*	208.7±8.35*	164.0±4.07*
Pae (80 mg/kg, i.g.)+ISO	79.91±3.43#	49.79±3.00#	145.4±6.20#	105.6±3.67#
DSS (160 mg/kg, i.g.)+ISO	73.73±3.67#	43.44±2.93#	132.7±8.12#	98.79±3.89#
Pae+DSS+ISO	56.73±3.48# @ $	32.44±3.38# @ $	87.91±6.45# @ $	83.92±4.20# @ $

Results are expressed as mean ± S.D. (n = 8).

* *P*<0.001 vs. Control;

# *P*<0.001 vs. ISO;

@ P<0.001 vs. Pae+ISO;

$ *P*<0.001 vs. DSS+ISO (one-way ANOVA).

### Effect of paeonol and danshensu on cTnI

**Figure 4:**
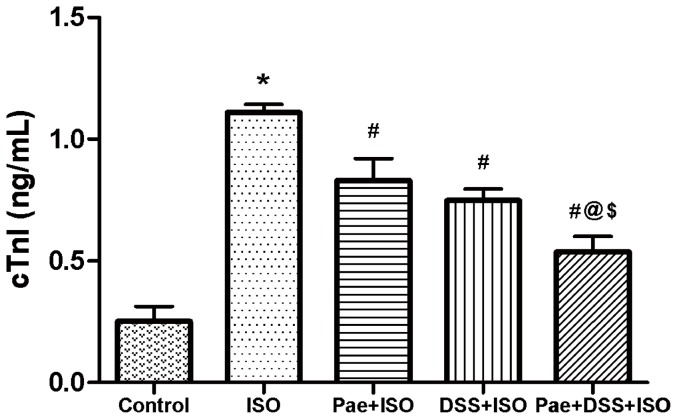
Levels of cardiac troponin I (cTnI) in the serum of control and experimental animals. Values are expressed as mean ± S.D. (n = 8). ^*^ *P*<0.001 vs. Control; ^#^ *P*<0.001 vs. ISO; ^@^ *P*<0.001 vs. Pae+ISO; ^$^ *P*<0.001 vs. DSS +ISO (one-way ANOVA).

Fig. 4 shows the level of cardiac troponin I (cTnI) in the serum of normal and isoproterenol-induced rats. Rats induced with isoproterenol showed significant (*P*<0.001) elevation in the levels of cTnI in serum compared to normal control rats. Pretreatment with paeonol and danshensu in combination showed significant (*P*<0.001) decrease in the levels of serum cTnI when compared to the ISO, Pae+ISO and DSS+ISO groups.

### Effect of paeonol and danshensu on lipid peroxidation and antioxidant enzymes

**Figure 5:**
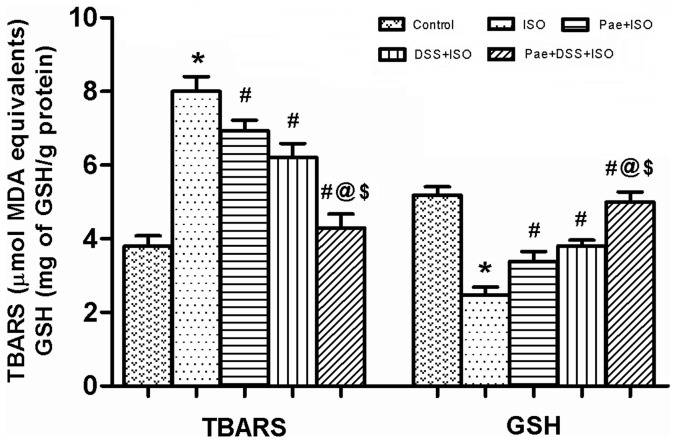
Levels of thiobarbituric acid reactive substances (TBARS) and glutathione (GSH) in the heart of control and experimental animals. Values are expressed as mean ± S.D. (n = 8). ^*^ *P*<0.001 vs. Control; ^#^ *P*<0.001 vs. ISO; ^@^ *P*<0.001 vs. Pae+ISO; ^$^ *P*<0.001 vs. DSS+ISO (one-way ANOVA).

The myocardial TBARS and GSH levels and activities of enzymic antioxidants such as SOD, CAT, GPx, GR and GST in the heart of normal and ISO-administered rats are shown in Fig. 5 and Table 3, respectively. Rats administered with ISO showed a significant (*P*<0.001) increase in the level of TBARS while there was a significant (*P*<0.001) decrease in GSH, SOD, CAT, GPx, GR and GST as compared to control groups. Combination of paeonol and danshensu along with ISO showed significant (*P*<0.001) protection than individual treatment groups in mitigating the parameters of oxidative stress.

**Table 3:** Effect of paeonol and danshensu on antioxidant enzymes activity in rats.

Group(s)	SOD	CAT	GPx	GST	GR
Control	91.19±3.52	87.33±2.70	8.65±0.64	12.28±0.51	11.94±0.67
ISO (85 mg/kg, s.c.)	55.57±3.65*	45.93±2.19*	3.82±0.48*	6.48±0.74*	6.61±0.67*
Pae (80 mg/kg, i.g.)+ISO	75.61±3.54#	64.15±3.18#	5.08±0.54 #	8.02±0.32#	7.57±0.46&
DSS (160 mg/kg, i.g.)+ISO	81.57±3.50 #	69.54±3.02#	6.40±0.49#	9.11±0.33#	8.45±0.45#
Pae+DSS+ISO	88.54±3.53 # @ ¥	83.93±3.02# @ $	8.04±0.65# @ $	11.67±0.41# @ $	11.29±0.74# @ $

Results are expressed as mean ± S.D. (n = 8).

* *P*<0.001 vs. Control;

& *P*<0.05 vs. ISO,

# *P*<0.001 vs. ISO;

@ P<0.001 vs. Pae+ISO;

¥ *P*<0.01 vs. DSS+ISO,

$ *P*<0.001 vs. DSS+ISO (one-way ANOVA).

Activity is expressed as units/mg protein for SOD, µmol of H_2_O_2_ decomposed/second/mg protein for CAT, µmol of GSH oxidized/min/mg of protein for GPx, µmol of CDNB conjugated/min/mg protein for GST and µmol of GSSG utilized/min/mg protein for GR.

### Effect of paeonol and danshensu on histological changes

**Figure 6:**
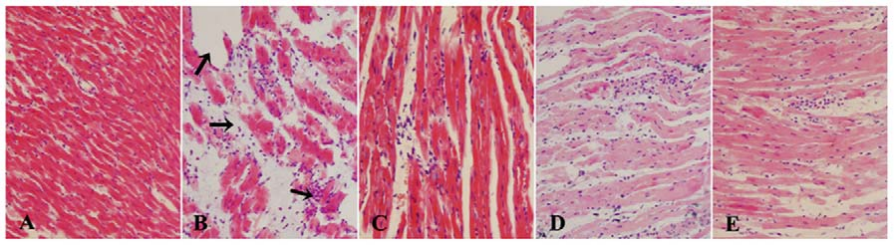
Effect of paeonol and danshensu on histopathological changes [(A) control, (B) ISO, (C) Pae+ISO, (D) DSS+ISO and (E) Pae+DSS+ISO] in heart tissue of control and experimental animals (Heart tissues were stained with hematoxylin and eosin and visualized under light microscope at ×200 magnification).

Histopathological observations of control rat heart showed a normal myofibrillar structure with striations, branched appearance and continuity with adjacent myofibrils (Fig. 6 A). ISO-induced rats revealed marked myofibrillar degeneration with infiltration of neutrophil granulocytes and interstitial edema (Fig. 6 B). The tissue sections from all treatment groups [Pae+ISO (Fig. 6 C), DSS+ISO (Fig. 6 D) and Pae+DSS+ISO (Fig. 6 E)] showed some infiltration with neutrophil granulocytes, interstitial edema and some discontinuity with adjacent myofibrils but the morphology of cardiac muscle fibers was relatively well preserved with no evidence of focal necrosis when compared to ISO-induced group. Combination of paeonol and danshensu showed a better morphology than individual treatment groups.

### Effect of paeonol and danshensu on protein expression of Nrf2, Bax, Bcl-2 and Caspase-3

**Figure 7:**
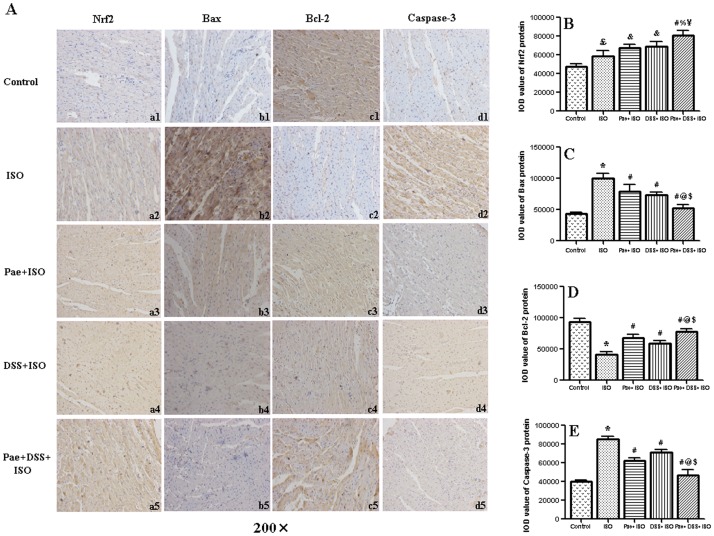
Graph A showing the representative immunohistochemical staining of Nrf2 (a1, a2, a3, a4 and a5), Bax (b1, b2, b3, b4 and b5), Bcl-2 (c1, c2, c3, b4 and c5) and Caspase-3 (d1, d2, d3, d4 and d5) expression in myocardium. Control group (a1, b1, c1, and d1), ISO group (a2, b2, c2 and d2), Pae+ISO group (a3, b3, c3 and d3), DSS+ISO group (a4, b4, c4 and d4) and Pae+DSS+ISO group (a5, b5, c5 and d5). Bar graph B, C, D and E showing the levels of Nrf2, Bax, Bcl-2 and Caspase-3 protein in myocardium, respectively, expressed as the integral optical density (IOD). Values are expressed as mean ± S.D. (n = 5). ^???^ *P*<0.05 vs. Control, ^*^ *P*<0.001 vs. Control; ^&^ *P*<0.05 vs. ISO, ^#^ *P*<0.001 vs. ISO; ^%^ *P*<0.01 vs. Pae+ISO, ^@^ *P*<0.001 vs. Pae+ISO; ^¥^ *P*<0.01 vs. DSS+ISO, ^$^ *P*<0.001 vs. DSS+ISO (one-way ANOVA).

The protein expression of Nrf2, Bax, Bcl-2 and Caspase-3 in the heart of normal and ISO-administered rats are shown in Fig. 7. Immunohistochemical analysis showed that ISO injection significantly (*P*<0.05 or *P*<0.001) increased the expression of Nrf2, Bax and Caspase-3 protein and decreased the expression of Bcl-2 protein in the myocardium when compared to control rats. However, by the treatment of combination of paeonol and danshensu, the expression of Bax and Caspase-3 or Bcl-2 and Nrf2 protein was down-regulated or up-regulated, respectively (*P*<0.001), when compared to ISO groups.

## Discussion and Conclusions

ISO in supramaximal doses induces morphological and functional alterations in the heart leading to subendocardial myocardial ischemia, hypoxia, necrosis, and finally fibroblastic hyperplasia with decreased myocardial compliance and inhibition of diastolic and systolic function, which closely resembles local myocardial infarction-like pathological changes seen in human myocardial infarction [30]. Generation of highly cytotoxic free radicals through auto-oxidation of catecholamines has been implicated as one of the important causative factor in isoproterenol-induced myocardial damage [31]. It has been reported that auto-oxidation of excess catecholamines results in free radical-mediated peroxidation of membrane phospholipids and consequently leading to permeability changes in the myocardial membrane, intracellular calcium overload and irreversible damage [6], [32].

In the present study, we have observed a significant increase in the heart weight and the ratio of heart weight to body weight in ISO-induced rats. Patel et al. [33] have reported that the observed increase in the heart weight in ISO-induced rats might be due to the increased water content, oedematous intramuscular space and extensive necrosis of cardiac muscle fibres followed by the invasion of damaged tissues by the inflammatory cells. Combined pretreatment of paeonol and danshensu significantly decreased the heart weight in ISO-induced rats.

The main criteria generally used for the definite diagnosis of MI is evolving pattern of Electrocardiograph (ECG)-abnormalities. ST-segment elevation reflects the potential difference in the boundary between ischemic and non-ischemic zones and consequent loss of cell membrane function. It was observed either in patient with acute myocardial ischemia [34] or in isoproterenol-induced myocardial infarction in rat [33]. In the present study, we noted a significant elevation of ST-segments in ISO-induced rats. The observed ST-segment elevation might be due to myocardial necrosis accelerated by ISO, which is in consistent with the observations of the earlier reports [33]. Pretreatment of paeonol and danshensu in combination markedly inhibited isoproterenol-induced ST-segment elevation, suggestive of its cell membrane protecting effects.

Extent of myocardial infarction is detected by direct staining with TTC dye, which forms a red formazan precipitate in the presence of intact dehydrogenase enzyme systems, whereas the infracted myocardium lack dehydrogenase activity and therefore fails to stain with it. Area of infarction may relate to leakage of dehydrogenases and loss of membrane integrity [35]. ISO-induced rats showed increase in myocardial infarct size with less TTC absorbing capacity, indicating significant leakage of dehydrogenases from the myocardium. This ISO-induced loss of dehydrogenases was counteracted by the co-administration of paeonol and danshensu, which a significant decreased level of infarct size was observed in Pae+DSS+ISO group, further supporting the better protection from cardiac damage of the combination.

Cytosolic enzymes namely CK-MB, LDH, AST and ALT serve as sensitive indices to assess the severity of myocardial infarction. Increased activities of these marker enzymes in the serum are indicative of cellular damage and loss of functional integrity and/or permeability of cell membrane [36]. Moreover, recent data have indicated measurement of cardiac troponin I (cTnI), a low molecular weight and contractile protein which is normally not found in serum, but released when myocardial necrosis occurs, may be even more significant in diagnosing acute MI and for risk prediction in subsequent infarction [37]. In the present study, a significant increase observed in the activities of CK-MB, LDH, AST, ALT and level of cTnI in serum of ISO-induced rats may due to the leakage of them from the heart as a result of necrosis induced by ISO. That is, the cardiac membrane becomes permeable or may rupture, due to deficient oxygen supply or glucose, thereby resulting in the leakage of enzymes and/or cTnI [38]. The combination of paeonol and danshensu seems to preserve the structural and functional integrity and/or permeability of the cardiac membrane and thus restricting the leakage of these indicative enzymes and cTnI from the myocardium, as evident from the markedly blunted levels of these enzymes and cTnI in Pae+DSS+ISO group when compared to the individual treatment groups, thereby establishing the cardioprotective effect of the combination of paeonol and danshensu.

Histopathological examination of myocardial tissue in control illustrated clear integrity of the myocardial cell membrane with no evidence of focal necrosis and inflammatory cell infiltration when compared to the ISO-induced heart. ISO-induced rats showed separation of cardiac muscle fibers and extensive infiltration of neutrophil granulocytes. The reduced inflammatory cell infiltration and normal cardiac muscle fiber architecture in Pae+DSS+ISO group further confirmed the cardioprotective effect of paeonol and danshensu.

Lipid peroxidation has been defined as the oxidative deterioration of polyunsaturated lipid. It occurs constantly at a low level in most cellular biological systems. Oxygen-derived free radicals can react with lipids, if not blocked by sufficient antioxidant molecules, to form lipid peroxides which do extensive damage [39]. Since the major constituents of biological membranes are lipids, their peroxidation can lead to cell damage and death. A significant increase in the levels of lipid peroxidation products in ISO-induced rats appear to be the initial stage to the tissue making it more susceptible to oxidative damage. Increased production of free radicals may be responsible for the observed membrane damage as evidenced by the elevated lipid peroxidation in terms of TBARS in the present study.

GSH is a tripeptide which has a direct antioxidant function by reacting with superoxide radicals, peroxy radicals and singlet oxygen followed by the formation of oxidized GSH and other disulfides. It plays an important role in the regulation of variety of cell functions and in cell protection against free radical mediated injury [5], [6], [40]. Thus, reduction in cellular GSH content could impair recovery after short period of ischemia. Depressed GSH levels may be associated with an enhanced protective mechanism to oxidative stress in MI. In this study, ISO administration was found to reduce the levels of GSH in cardiac tissue and the observation concurs with several earlier findings [20], [32], [33]. Decreased GSH levels might be due to increased utilization in protecting ‘SH’ containing proteins from lipid peroxides. Pre-co-treatment of combination of paeonol and danshensu decreased the levels of lipid peroxides (in terms of TBARS) while increased the level of GSH in the heart of ISO-induced cardiotoxic rats when compared to individual treatment groups. This shows the antilipid peroxidative effect of the combination of paeonol and danshensu against injury caused by free radicals. In this context, previous studies have shown danshensu and paeonol exerted significant GSH-elevating and MDA-scavenging effect in a PK-PD model of MI [41] and in a D-gal-induced cognitive impairment model, respectively [42].

Auto-oxidation of ISO produces quinones, which react with oxygen to produce superoxide anions and hydrogen peroxide, leading to oxidative stress and depletion of the endogenous antioxidant system. Free radical scavenging enzymes, such as SOD, CAT, GPx, GR and GST are the first line of cellular defense against oxidative injury, decomposing O_2_ and H_2_O_2_ before their interaction to form the more reactive hydroxyl radical [5], [40]. The equilibrium between the enzymatic antioxidants and free radicals is an important process for the effective removal of oxidative stress in intracellular organelles [43]. However, in pathological conditions like MI, the generation of ROS can dramatically upset this balance with an increased demand on the antioxidant defense system [5]. In this study, a significantly lower activity of the enzymes SOD and CAT was observed in heart of ISO-administered rats when compared to control rats, which is consistent with similar findings in number of earlier studies [20], [32]. The decrease in the activities of SOD and CAT might be due to their increased utilization for scavenging ROS and their inactivation by excessive ISO oxidants. The activities of GSH-dependent antioxidant enzymes (GPx, GST and GR) were declined in hearts ISO-administered. GPx offers protection to the cellular and subcellular membranes from the peroxidative damage by eliminating hydrogen peroxide and lipid peroxides. The lowered activities of GPx and GST in heart in ISO-administered rats may due to the reduced availability of GSH. Decreased activities of these enzymes lead to the accumulation of these oxidants and make myocardial cell membranes more susceptible to oxidative damage. Inactivation of GR in the heart leads to accumulation of oxidized glutathione (GSSG), which is an oxidized product of GSH. GSSG inactivates the enzymes containing SH-group and inhibits protein synthesis [44]. Pretreatment with the combination of paeonol and danshensu significantly increase in the activities of SOD, CAT, GPx, GR and GST when compared to individual treatment groups.

Some recently studies have reported the changes in the transcriptional regulation of antioxidant genes in humans and mouse models of disease [45], [46]. Evidence indicates that nuclear factor erythroid 2-related factor 2 (Nrf2) is the primary transcriptional regulator of a majority of the antioxidants including SOD, GPx, GST, GR and catalase [47]. Suh et al. have recently reported that a decrease of Nrf2 nuclear expression could be linked with down-regulation of transcription and translation products for GCLC and GCLM (rate-limiting enzyme for biosynthesis of GSH) [48]. Moreover, emerging evidence has revealed that Nrf2 signaling plays a key role in preventing oxidative cardiac cell injury in vitro [49]. In the present study, we observed elevated Nrf2 levels in the infarct border zone in the MI group. Significantly, the Nrf2 levels were further enhanced by paeonol and danshensu treatment. Thus, we presume that activation of Nrf2 related signaling is likely to be, at least partly, responsible for the protective effect of danshensu and paeonol against ISO-induced MI.

It is well established that both necrosis and apoptosis are involved in the acute stage of MI [4]. The interaction between pro-and anti-apoptotic proteins of Bcl-2 family integrates different death and survival signals to decide the fate of the cells [50]. During acute MI, the formation of ROS in infarction areas could exceed the anti-oxidative capacity hence initiate mitochondrial apoptotic signaling and activate Bax, a pro-apoptotic member of Bcl-2 family proteins [51]–[53]. In healthy cells, the majority of Bax is localized in the cytosol, but upon initiation of apoptotic signaling, activated Bax rapidly translocates to the mitochondria and undergoes a conformation shift, as well as interacts with Bak to form protein-permeable pores on mitochondrial membranes [52], [53]. This results in the release of cytochrome C and other pro-apoptotic factors from the mitochondria to the cytosol. Cytochrome C in turn stimulates Apaf-1 and Caspase-9 to form apoptosomes, and then activates Caspase-3, leading to cell death [52], [54]. On the contrary, the over-expression of anti-apoptotic Bcl-2 family protein has been shown to intercept the release of cytochrome C in response to a variety of apoptotic signals and therefore inhibits apoptosis. In our study, the Bcl-2 expression level was markedly decreased during the process of MI. However, paeonol and danshensu combinational treated groups inhibited the degradation of Bcl-2 protein in infarct regions. In contrast, an increased level of Bax and Caspase-3 protein was observed in MI groups. The upregulated Bax and Caspase-3 were inhibited by treatment with paeonol and danshensu in combination. Our data suggested that early treatment with danshensu and paeonol in combination might inhibit cadiocyte apoptosis, thus providing an anti-apoptosis strategy against MI damage.

In conclusion, our study reveals that the combination of paeonol and danshensu exerts significant cardioprotective effects against ISO-induced myocardial infarction in rats. This myocardial protective effect could be associated with the enhancement of antioxidant defense system through the activation of Nrf2 related pathway and anti-apoptosis through regulating Bax, Bcl-2 and Caspase-3. Further, the protective effect thus could provide experimental evidence to support the rationality of combinatorial use of traditional Chinese medicine in clinical practice.
